# Pharmaceutical excipients and the information on drug labels

**DOI:** 10.1016/S1808-8694(15)30976-9

**Published:** 2015-10-19

**Authors:** Aracy Pereira Silveira Balbani, Lucilena Bardella Stelzer, Jair Cortez Montovani

**Affiliations:** a PhD, voluntary professor; b Pharmacist and biochemist – University Hospital – Medical School of Botucatu (UNESP); c Associate professor of Otorhinolaryngology-head and neck surgery – Medical School of Botucatu (UNESP). Department of Otorhinolaryngology-Head and Neck Surgery – Medical School of Botucatu (UNESP)

**Keywords:** flavoring agents, pharmaceutical preservatives, pharmaceutic aids, parabens, drug labeling, tartrazine

## Abstract

**Aim:**

to evaluate the presence of preservatives, dyes, sweeteners and flavouring substances in 73 pharmaceutical preparations of 35 medicines for oral administration, according to drug labeling information about the excipients.

**Methods:**

35 medications were selected, both over-the-counter and prescription durgs, marketed in Brazil. The sample included: analgesic/antipyretic, antimicrobial, mucoregulatory, cough and cold, decongestant, antihistamine, bronchodilator, corticosteroid, antiinflammatory and vitamin medications. We collected data on 73 preparations of these drugs, according to drug labeling information regarding preservatives, dyes, sweeteners and flavourings.

**Results:**

Methylparaben and propylparaben were the most common preservatives found (43% and 35.6% respectively). The most common sweeteners were: sucrose (sugar) (53.4%), sodium saccharin (38.3%) and sorbitol (36.9%). Twenty-one medicines (28,7%) contained two sweeteners. Colourless medicines predominated (43.8%), followed by those with sunset yellow dye (FD&C yellow no. 6) (15%). Five products (6.8%) contained more than one colour agent. Tartrazine (FD&C yellow no. 5) was present in seven preparations (9.5%). Fruit was the most common flavouring found (83%). Labelings of drugs which contained sugar frequently omitted its exact concentration (77%). Of the four labelings of medicines which contained aspartame, two did not warn patients regarding phenylketonuria.

**Conclusions:**

Omission and inacuracy of drug labeling information on pharmaceutical excipients may expose susceptible individuals to adverse reactions caused by preservatives and dyes. Complications of inadvertent intake of sugar-containing medicines by diabetics, or aspartame intake by patients with phenylketonuria may also occur.

## INTRODUCTION

Excipient – or inactive constituent – is a substance without therapeutic power, used to guarantee stability, physicochemical and organoleptic properties of pharmaceutical products^1^.

Internal use medication excipients may be: preservatives, dyes, flavorings, sweeteners, thickeners, emulsifiers, stabilizers or antioxidants. They keep medication free from micro-organisms and proper for consumption for longer time spans, besides making them tasty and thus favoring treatment compliance^1^, ^2^.

The pharmaceutical industry uses thousands of excipients. A survey conducted in England found 3,816 of these substances in a sample of 12,132 medications. Because of the excipients, the drugs analyzed presented 710 different colors, 896 different flavors and 140 different odors^3^.

Most excipients are used in low concentrations, thus adverse reactions are rare^1^. However, they may trigger undesirable effects due to intolerance – a non-immune mechanism which leads to anaphylactic reactions and idiosyncrasies – or allergies – immune mechanism which may result in immediate or late hypersensitivity4. In clinical practice these reactions are commonly and mistakenly attributed to the medication active principle.

The pharmaceutical companies are obliged to discriminate all inactive ingredients in medication inserts^5^, ^6^. Notwithstanding, many health care providers do not read the insert or have no idea about excipients when prescribing drugs.

Following we present the characteristics and adverse reactions of some of the most used excipients in pharmaceutical formulas for oral intake.

### Preservatives

We highlight the parabens (methylparaben and propylparaben, for instance), used by the pharmaceutical, food and cosmetic industries since 1920. Parabens are large spectrum antimicrobial drugs, hydrosoluble, insipid, colorless and odorless^7^. With such characteristics they are broadly employed in the formulation of medications. Parabens concentrations vary, but hardly exceed 1%7.

Parabens are partially metabolized and becomes hydroxyparabenzoic acid, of which chemical structure is similar to that of acetylsalicylic acid. Although rare, parabens anaphylactic reactions may trigger urticaria and angioedema in individuals with salycilate intolerance^7^. The same symptoms may occur with the use of preservatives, such as benzoic acid and its salts (sodium, potassium and calcium benzoates)^7^.

Preservatives based on sulfur salts (calcium, potassium and sodium metabisulfites, for instance) have also been considered as causes of persistent rhinitis and chronic urticaria^8^.

### Dyes

Dyes may be organic or inorganic, natural or artificial. Examples of inorganic dyes are: Titanium dioxide and iron oxide^9^. Natural dyes are derived from plants or animals. Artificial dyes are made in the laboratory^4^.

Carmine red (# 4 red) is an example of a natural dye. It derives from carminic acid, extracted from the dry bodies of the Dactylopius coccus (cochonilha) female insect. Some cases of occupational asthma and food allergy have been attributed to carmine, of which physiopathological mechanism is IgE mediated^4^, ^10^.

Among artificial dyes we have the azo dyes – tartrazine yellow (FD&C # 5 yellow), dusk yellow (FD&C # 6 yellow), Bordeaux S (FD&C # 2 amaranth or red) and Ponceau 4R (FD&C # 4 red), erythrosine (FD&C # 3 red) and indigocarmine (FD&C # 2 blue)^4^.

Tartrazine yellow is found in a number of medications, cosmetic agents and foodstuff. Its chemical structure is similar to that of benzoate, salycilate and indomethacin, thus the possibility of crossed allergic reactions with these substances5. Moreover, tartrazine may trigger hyperkinesia and eosinophylia in hyperactive patients^5^. The occurrence of non-thrombocytopenic purpura is rare; however it does mean that tartrazine is able to inhibit platelet aggregation, similar to salycilates, sodium benzoate and sodium metabissulfite^11^.

Hypersensitivity to tartrazine happens in 0.6 to 2.9% of the population, with a higher incidence in atopic patients or those with intolerance to salycilates. The most common clinical aspects are: urticaria, bronchospasm, rhinitis and angioedema^11^. Despite the low incidence of tartrazine sensitivity in the general population, the drug companies are obliged, by force of law, to highlight a warning in the drug insert and package of the medications that have this dye^12^.

The dusk yellow dye (FD&C # 6) may also cause anaphylactoid reactions, angioedema, anaphylactic shock, vasculitis and purpura. There may be crossed reactions between dusk yellow, paracetamol, acetylsalicylic acid, sodium benzoate and other azo dyes^5^.

### Sweeteners

Liquid and chewable medications are usually very unpleasant to the taste, and sometimes it is necessary to combine different sweeteners in the same product in order to overcome this inconvenience^2^. The most used sweetener by the pharmaceutical industry are sucrose (sugar), its artificial surrogates (saccharin sodium, sodium cyclamate and aspartame)^13^ and sorbitol^5^.

Sucrose is of low cost, does not leave aftertaste and may act as preservative and antioxidant, it also enhances the viscosity of liquid medications. Its disadvantages are: crystallization during medication storage – which may clog the container lid – and use forbidden to diabetic patients^2^.

Kulkarni et al. (1993)^14^ assessed the liquid pediatric presentations of 499 medications, including: antitussive drugs, antimicrobial drugs, analgesics, antivomiting and antiparasitic drugs. They reported that 82% of the liquid formulations had sugar in them, and this makes them forbidden to diabetic children and fosters dental cavities.

People with lactose intolerance may present flatulence and diarrhea if they take medications that harbor this suggar^5^.

Aspartame, sodium cyclamate and sodium saccharin may induce hypersensitivity reactions, which cause urticaria, pruritus and angioedema^15^. There is the possibility of a crossed allergic reaction between saccharin, aspartame and sulfonamides^5^.

Cyclamates may cause photosensitization, eczema and dermatitis. They are no longer used as sweeteners in the USA since 1970, by a determination of the FDA - Food and Drug Administration^5^, because of its carcinogenic potential seen in laboratory animals, although this relationship between cyclamate use and cancer developing in humans have never been proved^16^.

Aspartame may cause renal tubular acidosis when used in large quantities^5^. Its use is counterindicated in patients with phenylketonuria because it has phenylalanine.

Sorbitol may cause osmotic diarrhea – usually followed by flatulence and abdominal pain; impairing the uptake of the medication active principle^5^.

### Flavorings

These ingredients are used to enhance the flavor of medications. They usually are industry secrets, thus not specified in medicine inserts.

Flavorings may be natural (essential oils extracted from plants and fruit natural flavors) or artificial (aromatic alcohol, aldehydes, balms, phenols, terpenes, etc). Benzil acetate, for instance, is a medication component with artificial cherry, peach, apricot and strawberry flavors^5^.

Adverse reactions to flavorings are rare, since these chemical compounds are employed in minute concentrations in medications^5^.

This study aimed at analyzing: a) the presence of preservatives, sweeteners, dyes and aromatizers in 73 drug preparations – liquid or in granules – of 35 oral use medications, and b) insert information about excipients.

## METHODS

We selected 35 oral use medications available in the Brazilian market. The sample included: analgesic/antipyretic, antimicrobial, mucolytic, antitussive, decongestants, anti-histamine, bronchodilators. Steroids, anti-inflammatory and vitamin supplements (Table 1). From June to September of 2004 we analyzed the inserts of 73 presentations of these medications from different commercial brands, prescription or over the counter drugs. For analysis purposes, both liquid and granulated presentations were chosen.

**Table 1:** Groups of medications analyzed, active principles and respective number of formulations included in the study.

Group	Active principles	# of formulations
Analgesic/antipyretic	Sodium dipirone	5
	ibuprofen	3
	paracetamol	5

Anti-inflammatory	Benzydamine chloridrate	1
	nimesulide	2

Anti-histaminic	Cetirizine chloridrate	1
	Epinastine chloridrate	1
	loratadine	1
	desloratadine	1
	ebastine	1
	dexchlorpheniramine maleate	1

Antimicrobial	amoxicillin 600mg, potassium clavulanate 42.9mg	1
	amoxicillin 50mg, sulbactam 50mg	1
	azithromicin	2
	monohydrated cephadroxyl	2
	monohydrated cefprozil	1
	trimethropin, sulphametoxazole	3
	Sultamicillin tosilate	1

Antitussive	dropropizine	3
	dropropizine, paracetamol, diphenydramine chloridrate, de pseudoephedrine chloridrate	2
	levodropropizine	5

Bronchodilators	acebrophylin	4
	bambuterol chloridrate	1

Steroids	prednisolone	3
	dexchlorphenyramine maleate, betamethasone	1

Oral decongestants	bronpheniramine, phenylephrin	2
	paracetamol, phenylephrin, chlorphenamine	2
	loratadine, pseudoephedrine sulphate	2
	azatadine maleate, pseudoephedrine sulphate	1

Mucolytic	carbocystein	6
	ambroxol chloridrate	3
	acethylcystein	1
	Hedera helix dry stratum	1

Vitamin supplements	polyvitamins	2
	Ascorbic acid	1

Total		73

Insert data were noted on the presence of preservatives, sweeteners, dyes and aromatizers in the formulations. In cases of doubts on the insert information we called the Customer Care Service of the respective pharmaceutical companies.

## RESULTS

Of the 73 pharmaceutical formulations analyzed, 31 were oral drops or liquid solutions (42.5%), 26 were syrups (35.6%), 13 were oral suspensions (17.8%) and three were in granules (4.1%).

All inserts mentioned the formulation excipients in details, except for a carbocystein presentation in drops, sold over the counter.

### Preservatives

Table 2 lists insert information about the presence of preservatives in the medication formulas. The total adds to more than 100% because many of the drugs have more than one preservative. The most frequent ones were: methylparabenzene, found in 33 formulations (45.2%), propylparaben, in 26 formulations (35.6%), sodium benzoate, in 24 presentations (32.8%) and sodium metabisulfite, in eight formulations (11%)

**Table 2:** Preservatives found in the liquid pharmaceutical formulations analyzed (n=73).

Preservatives	# of formulations (%)
Methylparaben	33 (45,2%)
Propylparaben	26 (35,6%)
Sodium benzoate	24 (32,8%)
Sodium metabisulfite	8 (11%)
Benzoic acid	4 (5,4%)
Hydroxyparabenzoate	4 (5,4%)
Potassium sorbate	2 (2,7%)
hydroxyparabenzoic acid	1 (1,3%)
Does not have preservatives**	2 (2,7%)

** Complex B polyvitamins in drops and acethylcystein in granules.

### Sweeteners

The sweeteners used in the formulations studied were sucrose (sugar) in 39 drugs (53.4%), sodium saccharin in 28 (38.3%), sorbitol in 27 (36.9%), sodium cyclamate in 19 (26%) and aspartame in four (5.4%).

Twenty one medications (28.7%) had two sweeteners; seven (9.5%) had three sweeteners and three (4.1%) had four sweeteners. Of the 26 pharmaceutical formulations in syrup-type, 18 (69.2%) had sugar in it.

Among the inserts of 39 medications which had sugar in them, only nine (23%) specified its concentration, which varied from 0.349 g/ml (in a carbocystein cough syrup) to 0.72 g/ml (in an oral solution of sodium dipirone).

We contacted the Customer Care Service of a pharmaceutical company to clarify the sugar concentration in two of their products and received the information requested. According to the attendant, the sugar concentration in the medication is not mentioned in the insert “in order to prevent the competition to copy the compound”.

The inserts of the medication containing sugar varied as to the information given to diabetic patients. Two commercial brands of the sodium dipirone oral solution alerted that the product should not be used by diabetic patients because it had sugar in it – they warned customers that the product was counter-indicated for diabetic patients, without specifying the reasons why. Three carbocystein formulations (adult and pediatric solutions and pediatric drops), of the same commercial brand, informed in the insert that “diabetic patients should consult a physician” and “be carefully monitored” during treatment. One formulation of an antitussive syrup with dropropizine warned that “diabetic patients should consider the sugar content present in each formulation”. One of the commercial brands of loratadine decongestant syrup with pseudoephedrine did not mention its use by diabetic patients.

Considering the four presentations with aspartame, a cefprozil suspension required “special attention with phenylketonuric patients”, informing that each 5ml of reconstituted suspension had 28mg of phenylalanine. A suspension of amoxicillin 600mg with clavulanate potassium 42.9mg/ml recommended “careful with phenylketonuric patients”, informing that each 5ml of medication had 7mg of phenylalanine. The insert of a commercial brand of carbocystein (in granules - adult and pediatric formulations) omitted the information about its use by phenylketonuric patients.

### Dyes

Among the formulations analyzed, 32 (43.8%) did not have dyes. The dyes present in the other medications are listed on Table 3. Five products (6.8%) had more than one type of dye.

**Table 3:** Dyes found in the pharmaceutical formulations analyzed (n=73).

Dyes	# of formulations (%)
Dusk yellow (FD&C # 6)	11 (15%)
Tartrazine yellow (FD&C #5)	7 (9,5%)
Erythrosine	5 (6,8%)
Ponceau 4R red	4 (5,4%)
Caramel	3 (4,1%)
Red # 40	3 (4,1%)
Food red*	3 (4,1%)
Bordeaux S red*	2 (2,7%)
Quinoline yellow	2 (2,7%)
Yellow # 10	2 (2,7%)
Blue # 1	2 (2,7%)
Red # 10	1 (1,3%)
Iron oxide	1 (1,3%)

* Data not specified in four inserts, obtained through the manufacturer customer care center.

The inserts of a commercial brand of levodropropizine (formulations: pediatric syrup and drops), acetylcysteine (drops) and acebrophylline (formulation: pediatric syrup) did not specify which was the red dye employed in the formulas. According to their customer care service, the first two presentations had the Bordeaux S red dye, and the latter had the food red.

Tartrazine yellow was found in seven drug presentations (9.5% of the total): a) sodium dipirone in drops; b) paracetamol in drops; c) Benzydamine chloridrate in drops; d) pediatric decongestant liquid with bronpheniramine and phenylephrin; e) antitussive adult liquid with dropropizine, paracetamol and diphenydramine and pseudoephedrine chloridrates and f) antitussive – syrup and drops – based on levodropropizine. All the inserts included a warning about tartrazine, mandatory by Resolution RE # 572 from 04/05/2002 of the Agência Nacional de Vigilância Sanitária (Brazilian Equivalent of the American FDA)^12^.

### Aromatizers

Sixty one medications (83%) had the smell of fruits; six (8%), of tutti-frutti; four (5%) smelled of vanilla, four of caramel and 16 (22%) had other smells or essences (anise, cinnamon, gum, chocolate, egg, cassis, liquor, honey, menthol, rum, “forest smell”, “essence”, “half by half essence”, “sweet aroma” or “non-specified natural and artificial aromas).

Sixteen medications (21.9%) had two essences; seven (9.5%) had three essences.

## DISCUSSION

Kumar et al. (1993)^5^ analyzed the information on excipients present in the inserts of 102 drugs, and they saw that the data on preservatives, dyes and sweeteners present on the medications were omitted, in 35%, 20% and 10% of the inserts, respectively. In Brazil, Oliveira; Storpirtis (1999)^1^ assessed the inserts of 57 medicines and observed that only 40% had detailed information on the drug composition. In this paper we noticed that only one of the 73 inserts analyzed – of an over the counter medicine, for pediatric use, did not list the medicine excipients.

Notwithstanding, sugar content was omitted in the inserts of 77% of the medicines that had the sweetener. It is very likely that we had two factors contributing to this fact: 1. the industrial secret around the product composition and 2. the voluntary character of the information, since quantifying inactive ingredients is not required by law^6^.

Inserts belonging to products sweetened by aspartame did not mention the precautions to be taken by phenylketonuric patients, and four inserts (5.4%) did not specify the dye in their formulation. We had to call the customer care center of the drug companies in order to obtain the information. On the other hand, the inserts of all the seven medications which had the tartrazine yellow dye included the mandatory warning about the dye, complying with the legislation in effect^12^.

The omission and imprecision of some insert information about inactive ingredients expose susceptible individuals to the risk of having adverse reactions to some preservatives and dyes. Moreover, there may be complications arising from the injudiciously use of drugs with sugar by diabetic patients, or of drugs sweetened by aspartame by phenylketonuric patients. For better safety, we believe it to be necessary to specify, in the drug insert, the type of dye used and, for the drugs that bear sugar or aspartame, stress the fact that diabetic and phenylketonuric patients, respectively, may or may not consume them. This is specially important in the inserts of over the counter medications, since we presuppose the consumer will use these drugs without the guidance of a physician.

We observed some differences in the frequence of use of dyes in relation to the study by Kumar et al. (1993)^5^. In that sample, the most encountered dyes were # 40 red (41% of the medicines) and dusk yellow (26%). Most of the medications included in this study did not have dyes (48%) and those which had them were colored by dusk yellow (13%).

We highlight the fact that 13 commercial brands of antihistamine drugs analyzed by Kumar et al. (1993)^5^ had dyes, while the six formulations we assessed were colorless. It is possible that in the last decade the pharmaceutical industry has decided to suppress the use of dyes in many formulations, in order to reduce adverse reactions. On the other hand, only one of 102 presentations assessed by Kumar et al. had tartrazine yellow (0.98%)^5^, while in our sample this dye was found in 9.5% of the medicines. Considering tartrazine adverse reaction risks, we believe it to be of benefit for the Brazilian pharmaceutical industry to replace it by natural dyes, such as sugar beet red^18^.

The most used preservatives in the medicines we assessed were methylparaben and propylparaben (45.2% and 35.6%, respectively). We highlight that only two formulations (complex B polyvitamin drops and in granules acetylcysteine) did not have preservatives.

The sweeteners we found more frequently were: sucrose (sugar), in 53.4% of the medicines, sodium saccharin, in 38.3% and sorbitol in 36.9%. Thus, similar to Kulkarni et al. (1993)^14^, we also noticed that most of the formulations in syrup (69.2%) had sugar. It is important that physicians educate their patients – specially the pediatric population – to brush their teeth after ingesting sucrose-containing drugs, in order to prevent cavities. For chronic diseases that require long use of medicines, it is preferable to prescribe formulations in drops or artificially sweetned^2^.

The most found aromatizers in the pharmaceutical formulations studied were those resembling fruits (83%), among the large variety of essences used in the products. It is certain that these ingredients have a great psychological impact over consumers. Based on this we see that aromatizers are fundamental for the public to accept pharmaceutical products.

## CONCLUSIONS

Analyzing excipient information in the inserts of 73 formulations of 35 medicines used orally, we noticed that:

1. One insert (1.3%) did not list the inactive ingredients in the product.
2. The most commonly found preservatives were methylparaben (45.2%) and propylparaben (35.6%).
3. Drugs without dyes (43.8%) and those dyed by dusk yellow prevailed – yellow FD&C # 6 - (15%). Tartrazine (yellow FD&C # 5) was found in seven formulations (9.5%).
4. The most frequently used sweeteners were: sucrose (sugar), in 53.4% of the medicines, sodium saccharin (38.3%) and sorbitol (36.9%).
5. The most commonly found aromatizers were the fruity ones (83%).
6. Thirty inserts of sugar loaded medications (77%) did not state the amount of sweetener present in the product. Two of the four inserts of aspartame loaded medicines did not mention the precautions about their use by phenylketonuric patients. Four inserts (5.4%) did not specify the type of dye used in the formulations.
